# A Study of Electricity, with the View of Comprehending Its Application in Dentistry

**Published:** 1889-12

**Authors:** Howard E. Roberts

**Affiliations:** Philadelphia


### International Dental Journal.

Vol. X. December, 1889. No. 12.

### Original Communications.[1]

### A STUDY OF ELECTRICITY, WITH THE VIEW OF COMPREHENDING ITS APPLICATION IN DENTISTRY.[2]

BY HOWARD E. ROBERTS, D.D.S., PHILADELPHIA.

In preparing this paper for your consideration I have found it difficult to express my ideas without defeating the object for which it is written. To those who have given the subject much study, or who are practical electricians, my paper will be of little interest, but as it is to those who are not so well posted in the subject that I wish to address my remarks, I trust you will pardon me if I repeat what you are already familiar with. It is difficult to understand and remember if we do not know and see the reason why; therefore, I will try to explain some of the laws and define some of the terms used, making use of as few technical terms as possible, and defining those I have to use.

In many ways electricity may be compared to a stream of water, and, as we can see and measure the water, it makes it easier to understand and appreciate the laws which govern the electrical

[1] The editor and publishers are not responsible for the views of authors of papers published in this department, nor for any claim to novelty, or otherwise, that may be made by them. No papers will be received for this department that have appeared in any other journal published in this country. The journal is issued promptly on the 15th of the month.

[2] Read at the twenty-first annual meeting of the Pennsylvania State Dental Society, held at Cresson, Pa., July 30, 1889.

current. One difficulty which many have in trying to understand many of the terms used in connection with electricity comes from the impossibility of being able to see and handle,—you might say there is nothing tangible to work from.

In order to speak intelligently about electric currents, there are three terms or words the meaning of which it is necessary to understand. They are the Volt, the Ohm, and the Ampère, and they are so related that by Ohm's law, if any two are known we can determine the third. The volt is the unit of pressure, the ohm is the unit of resistance, and the ampère is the unit of volume or quantity.

In defining these terms I will first take the unit of resistance. If it were water or steam we were considering, we could say that the unit of resistance would be the resistance offered by a pound weight, and with water flowing through a pipe the resistance would be the friction of the water against the sides, which would be represented by so many pounds. Every wire or conductor, or non-conductor either, offers a certain resistance to the passage of a current of electricity, and the resistance which is offered by a column of pure mercury 106 centimetres long by one square millimetre cross-section at 0° C. has been accepted as the unit of resistance, and has been called the ohm. One mile of ordinary telegraph wire has about 13 ohms resistance. As we have a unit of resistance, we must have a unit of pressure to overcome that resistance, and it is called the volt. One volt will overcome a resistance of one ohm. With water we would say there was a pressure of so many pounds, and with electricity it is a pressure of so many volts; in either case it is a pressure to overcome a resistance. The ampère is the unit of volume or quantity; with water it would be the gallon, but we cannot measure electricity as we would water, though we can measure the effect that a given current will produce in depositing a given metal from its salt held in solution, and the deposition is directly proportional to the current.

The quantity of electricity that will pass in one second through a resistance of one ohm when the pressure is one volt has been taken as the unit of quantity, and is called the ampère. One ampère will deposit $\frac{17,253}{1,000,000}$ of one grain of silver in one second. It is said that one volt will overcome a resistance of one ohm, and it is a little difficult to understand or catch the exact meaning of it. I think a pipe carrying water will help us to understand. Suppose we have 1000 feet of pipe which we can lay perfectly level; now turn up both ends and fill the pipe with water; if either end is elevated, say one inch, the water will flow slowly from the other. An elevation of one inch would represent a pressure of about $\frac{1}{24}$ of one pound; now increase the elevation of that end to two feet, which will give about one pound pressure, and we will find the water to flow about twenty-four times faster. If, on measuring the water flowing from the pipe, we found one gallon to flow in one second, we would say that one pound would overcome the resistance of the pipe, so that one gallon of water will be discharged in one second, and one volt will overcome the resistance of one ohm, so that one ampère will be discharged or flow in one second, which is one way of writing "Ohm's law."

If we increase the voltage while the resistance is kept constant, we will increase the number of ampères, or, if the resistance is increased with a constant voltage, the number of ampères will be decreased, and the increase or decrease will be in direct proportion. Divide the volts by the ohms,—that is, the pressure by the resistance,—and the quotient will be the ampères.

By the "difference of potential" is meant the difference of pressure or level, and the earth is taken as zero potential in the same way that the ocean is the zero for water level, the current flows from the higher to the lower potential; we say there is a difference in potential of so many volts.

I have not given as much attention to the galvanic battery as I have to some other parts of the subject; however, I may be able to give you some ideas that may help you to explain some things which you do not now quite understand.

I believe there are two theories about the generation of electricity with the battery. One is that it is due to chemical action, and the other is that it is simply the contact of different metals or substances, and that it is the contact which produces the chemical action. The plates of a battery are supposed to be of different potentials, and, as the current falls from one to the other through the external circuit, the battery fluid serves the purpose of a pump, as it were, to raise it from the lower to the higher potential, so that it can again fall, and thus keep up a continuous circulation.

How much power can be gotten out of a battery, and how is the best way to arrange the cells, are questions frequently asked. The power of one horse is supposed to be able to lift thirty-three thousand pounds one foot in one minute, or five hundred and fifty pounds in one second. The electrical equivalent of the horse-power is seven hundred and forty-six watts. A watt is one volt multiplied by one ampère,—a volt ampère, as it is called,—and any combination of volts and ampères which when multiplied will give seven hundred and forty-six will represent one horse-power.

For instance, the horse-power may be one ampère and seven hundred and forty-six volts, or it may be one volt and seven hundred and forty-six ampères, and one volt and one ampère will be $\frac{1}{746}$ of one horse-power.

Ohm's law is one of the most important of electrical laws, and is the key to the distribution and use of electricity. The law is, "The strength of a current in a wire or conductor is in direct proportion to the difference of potential between its ends, and inversely proportional to its resistance," and the units are so chosen that one volt will overcome the resistance of one ohm, so that one ampère will flow in one second. You may divide or multiply and split your current and conductor as you please, but the law will hold good.

Let us now consider the power of a battery-cell. Take a simple plunge-battery having two carbon plates and one zinc, it will give about two volts and, we will say, ten ampères on short circuit,—that is, with no external resistance. Multiply two by ten, and we have twenty watts; divide 746 by 20, and we have $\frac{1}{37.3}$ of one horse-power. But before we can use that power, we have to introduce a transformer, say a motor or mallet, which will have some resistance, say one ohm.

The cell that will give ten ampères with two volts must have an internal resistance of $\frac{1}{5}$ ohm, for the volts divided by the ampères will give the resistance. If we add the external and internal resistance, we get $1\frac{1}{5}$ ohm; divide the two volts by that, and we have $1\frac{2}{3}$ ampères, which is multiplied by the two volts, giving $3\frac{1}{3}$ watts, which is a very small part of a horse-power, being only $\frac{1}{223}$.

We will now take six cells of the same kind and arrange them in series,—that is, with the carbon of one cell joined to the zinc of the next, etc. The external resistance is the same, but the internal is six times as great, or $1\frac{1}{5}$ ohms; add the external and internal resistance ($= 2\frac{1}{5}$ ohms), and divide the volts, which would be 12, by it, and we have $5\frac{5}{11}$ ampères of current; multiply 12 volts by $5\frac{5}{11}$ ampères, and we get $65\frac{5}{11}$ watts; divide 746 by that, and we get a little less than $\frac{1}{11}$ horse-power.

In connecting the cells in series you increase the voltage, but the ampères remain the same; while with the cells in parallel,—that is, all the carbons connected to one pole and the zincs to the other,—you increase the ampères by decreasing the internal resistance, but the voltage is the same as for one cell. Though the watts of the battery would be the same on short circuits in either case, the power through the resistance of one ohm with the cells in parallel would be but little better than for the single cell.

In regard to the arrangement of the cells to give the best results: it is said, to so arrange them that the internal and external resistance shall be the same will enable you to get the greater amount of work from the battery, and therefore that should be the best and most economical arrangement. The idea is that, as the resistances are equal, there would be an equal amount of heat used in either circuit, or fifty per cent. of the total energy, and that with a perfect transformer of power you could never realize more than half the power developed by the battery. If you want to do the greatest amount of work in the shortest time, I believe that arrangement of cells will do it, but I think it is not the most economical arrangement.

In every case where electrical energy is converted into mechanical there is generated what is called a counter-electromotive force. If this counter-electromotive force equals the electromotive force of the battery, there would be no current flowing; consequently there would be no heating, and no work would be done. Electromotive force means the pressure of the current, or the number of volts given by the battery, and is generally written an E. M. F. of so many volts. The heating of the circuit is in proportion to the square of the current. The counter-electromotive force acts as a resistance, inasmuch as it reduces the current, and it should be as great as possible, to allow enough current to flow to do the work. Make the internal resistance of the battery as small as possible,—that is to say, use large carbon and zinc surfaces. The heat in the cells is wasted; you can only use the current in the external circuit.

In the calculation of the battery power, it is the electrical and not the mechanical power you get; there is always a loss in the conversion of energy.

In converting the heat-energy of coal into mechanical, in the best and largest engines we cannot recover more than twenty per cent., while the locomotive engine, I believe, uses about eight per cent. With a good motor about ninety per cent. of the electrical may be converted into mechanical energy.

In regard to the storage battery and its care I feel that I know little, but as there may be some here who know less, I will try to define it.

Take two plates of lead and place them in dilute sulphuric acid (ten-per-cent. solution), and pass a current from two or more cells through them for a short time; upon disconnecting the cells and connecting the lead-plates you will get a current in the opposite direction, which will only last for a short time, though it may be of large volume; if the plates are charged and discharged a great many times in opposite directions, which is called forming the plates, they get a spongy surface upon them, when they are capable of receiving a large amount of electricity and returning a large portion of it. (Planta.) The forming process took several weeks, and was very tedious. Now the plates are generally coated or filled with oxide of lead in some manner, so that once charging forms the plates.

The storage-cell gives about 2.25 volts when freshly charged, which soon falls to 2, and is held at that for the greater part of the discharge. You cannot recover all of the electricity which is put into the cell in charging, but you can recover in a useful form a current which would be too small to use either with a lamp, cautery, or motor. The storage-cell may be charged with a battery of gravity-cells which would give a very small current. But it would take many hours' charging to get one hour's work, and the rate of discharge will depend upon the resistance of the circuit.

Before considering the use of the electric-light current in our offices, I think it would be well to understand a little about the generators of the current or the dynamos.

Professor S. P. Thompson's definition of a dynamo-electric machine is, "A machine for converting energy in the form of mechanical power into energy in the form of electric currents, or *vice versa*, by the operation of setting conductors to rotate in a magnetic field, or by varying a magnetic field in the presence of conductors." The generators may be divided into two classes,—that is, those giving a continuous current, or a current flowing in one direction; and those giving an alternating current,—that is, the current that flows first in one direction and then in the other, the alternations being very rapid.

The alternating current is not adapted to run the mallet, neither are there motors for that circuit very well adapted for our use; therefore I will not consider that current or its generators further.

The continuous current generators may be again roughly divided into those giving currents of high and those of low potential or voltage, though this is a comparative division. The dynamo to give a high potential current has many turns of rather fine wire on its armature, and is intended to give a current of small volume and great pressure, while the low potential dynamo gives a large current at low pressure, and has few turns of large wire or copper bars on its armature. One generator may give 10 ampères at 1000 volts, or 10,000 watts, and another would give 100 ampères at 100 volts, which also would be 10,000 watts, requiring the same power to drive them, and giving out the same power. The dynamo giving 100 volts would be called a low potential machine, and you could handle the wires without danger and with but little care, while a shock from the 1000-volt machine would probably prove fatal; the wires would be dangerous things to fool with, and they must be handled with great care.

The question is naturally asked, Why is one machine, or the current from one machine, dangerous, and the other harmless, when they both represent the same power? The answer comes from Ohm's law in this way. The resistance of the body from hand to hand with a dry skin is something like 3000 ohms, with the skin wet and making good contact, it will be in the neighborhood of 1500 ohms. If we have 100 volts with a resistance of 3000 ohms, we would only get one-thirtieth of an ampère through the body, while with 1000 volts there would be ten times as much, or one-third of an ampère. It is the ampères which do the work, and the volts are only necessary to overcome the resistance of the circuit, so that the required number of ampères can flow. You can take a spark from a Holtz machine which may be 1,000,000 volts without danger, because the current is infinitesimal.

The dynamo does not generate electricity, but it does generate a difference of potential; the amount of electricity flowing will depend entirely upon the resistance of the circuit, if the difference of potential is kept constant.

The arc light is generally placed on a circuit of high potential, while the incandescent is used with a low potential. The arc-light current, or the current of high potential, should *never* be introduced into the dentist's office for the purpose of using it to run the engine or mallet, or for any other purpose where the conductors have to be handled or brought in contact with either the patient or operator. The current could be handled and used all right if it were possible to be certain that the insulation of the wires would never fail, but, as that is an impossibility, no one has a right to expose himself, and particulary his patient, to a possible injury.

The incandescent current of 110 volts is higher than we want, but it is impossible to hurt your patients with it without making a special effort, and even then it would be very difficult to do more than burn them some. In using the incandescent current to run the engine an electric motor is used, which is so constructed that the full current can pass through without any external resistance being introduced, when the motor will run at its maximum speed; it will also generate a counter E. M. F. nearly equal to the E. M. F. of the dynamo.

To regulate the speed, there is used what is called a resistance-box, in which is wound a number of coils of German-silver wire, and by pressing a lever with the foot more or less of the wire is thrown into the circuit, and in that way regulating the amount of current passing through the motor, and consequently the speed. It is theoretically possible to get a better method of regulating the speed of the engine, but whether it is practical, I have not yet determined.

If a motor wound for use with a battery were put in an incandescent circuit without any external resistance, there would be so much current pass through, on account of its low resistance, that it would be burned out; and if resistance were added until only the number of ampères for which the motor was wound could pass, there would be a great many watts wasted; and if the watts were reduced to the watts of the battery, the motor would probably not go at all. The motor has to be wound for the number of volts of the circuit on which it is to be used.

In using the incandescent circuit for running the mallet there has to be a very decided change in the potential,—that is, it must be reduced.

Where there is a potential difference of about 30 volts, it is possible to produce an electric arc, and when the arc is formed the current is not broken. As the action of the mallet depends upon the interruption or breaking of the current, the mallet would not work where there was a potential difference of 30 volts, because an arc would be formed at the interrupter.

I believe the mallet will work best with from 4 to 6 volts, or say three cells, so that the volts ought to be reduced to about that number. Adding resistance will reduce the current, but not the volts; therefore some other means has to be found.

In the apparatus gotten out by the S. S. White Dental Manufacturing Company, resistance is introduced in the form of three lamps, so as to reduce the current to about two ampères; the current is then divided, part passing through the mallet, and the rest through a variable resistance which is less than the resistance of the mallet, so that most of the current passes through the variable resistance. The mallet works nicely, the arrangement is simple, and there is very little to get out of order. The current used represents about one-third of a horse-power, a small part only of which is used in the mallet. When the current is measured by meter and charged for accordingly, one-third of a horse-power costs less than three cents an hour. A motor of sufficient power to drive the engine should not cost as much per hour.

The electric motor is the reverse of the dynamo, and depends for its power upon magnetic attraction and repulsion, or upon the attraction and repulsion of a magnet for a conductor carrying an electric current, or both.

The amount of magnetism induced in an iron bar will depend upon the number of ampères passing around it, and is independent of the volts. With a given bar of iron the magnetism will be the same if 100 ampères is passed once, or 1 ampère 100 times around it.

Any one who wishes to use the incandescent current in connection with dentistry should always remember Ohm's law, should give study enough to the subject to understand the principle upon which the appliance they use is founded, and understand its construction. The use of electricity in dentistry is in its infancy, and there is room for improvement in the existing appliances and for new ones.

I think there are few who, having used the incandescent current and understanding its use, would be willing to give it up.

